# Association between Washing Residue on the Feet and Tinea Pedis in Diabetic Patients

**DOI:** 10.1155/2015/872678

**Published:** 2015-02-22

**Authors:** Kimie Takehara, Ayumi Amemiya, Yuko Mugita, Yuichiro Tsunemi, Yoko Seko, Yumiko Ohashi, Kohjiro Ueki, Takashi Kadowaki, Makoto Oe, Takashi Nagase, Mari Ikeda, Hiromi Sanada

**Affiliations:** ^1^Department of Nursing Administration, Graduate School of Medicine, The University of Tokyo, 7-3-1 Hongo, Bunkyo-ku, Tokyo 113-0033, Japan; ^2^Department of Gerontological Nursing/Wound Care Management, Graduate School of Medicine, The University of Tokyo, 7-3-1 Hongo, Bunkyo-ku, Tokyo 113-0033, Japan; ^3^Department of Dermatology, Tokyo Women's Medical University, 8-1 Kawada-cho, Shinjuku-ku, Tokyo 162-8666, Japan; ^4^The University of Tokyo Hospital, 7-3-1 Hongo, Bunkyo-ku, Tokyo 113-0033, Japan; ^5^Department of Metabolic Diseases, Graduate School of Medicine, The University of Tokyo, 7-3-1 Hongo, Bunkyo-ku, Tokyo 113-0033, Japan; ^6^Department of Advanced Nursing Technology, Graduate School of Medicine, The University of Tokyo, 7-3-1 Hongo, Bunkyo-ku, Tokyo 113-0033, Japan

## Abstract

Tinea pedis (TP) may lead to the development of foot ulcers in diabetic patients; thus, its prevention in diabetic patients is important. TP occurs after dermatophytes on the skin scales of TP patients attach to the feet. Therefore, it is necessary to remove the scales and dermatophytes, and this can be performed using various methods, including foot washing. This study aimed to objectively examine the association between the presence of TP and foot-washing habits. We included 33 diabetic patients, and, of these, 17 had TP. The presence of washing residue on the feet was determined by applying a fluorescent cream to the participants' feet, and images of the feet were captured under ultraviolet light before and after foot washing. Our results showed that diabetic patients with TP had higher levels of washing residue on their feet than those without TP. The importance of washing feet to prevent TP needs to be emphasized through educational programs for diabetic patients. Furthermore, the development of an effective foot-washing technique is essential.

## 1. Introduction

Diabetic foot is defined as an infection, ulceration, or destruction of the deep tissues of the foot that is associated with neuropathy and/or peripheral arterial disease in the lower extremities of people with diabetes [1]. This complication associated with diabetes can result in lower leg amputation and can reduce the quality of life [2, 3]. Tinea pedis (TP) is one of the causes of diabetic foot; therefore, preventing TP in diabetic patients is important. However, depending only on patient education to prevent diabetic foot is not adequate, and patients do not receive the necessary foot care education for preventing TP [4].

TP occurs after dermatophytes on the skin scales of TP patients attach to the feet. Dermatophytes grow hyphae and invade corneocytes, and they live parasitically within the horny layer [5, 6]. However, it takes at least 12 h for the dermatophytes to invade the corneocytes [7]; therefore, a dermatophyte infection can be prevented by removing them before they invade the horny layer, and washing the feet is one removal method. A previous study investigated the effects of foot washing on TP prevention in nondiabetic subjects and reported that the number of dermatophytes adhering to the feet reduced after the feet had been washed with soap [8]. In another study, fungal elements that had been collected from the heel area of the human foot were applied to the surface of the horny layer, and they were incubated* in vitro*. This study showed that fungal elements can be removed from the skin by washing feet with soap, and this was possible even after the fungi were incubated for two days [9]. However, these studies did not assess fungal attachment and foot-washing behavior during daily life. Indeed, few studies have investigated foot-washing behaviors. One study suggested that daily foot washing can be effective for preventing onychomycosis in diabetic patients [10], and because onychomycosis is associated with TP [11, 12], these findings may suggest that patients with TP may also have a higher amount of washing residue after they wash their feet.

To evaluate washing behaviors, some studies used fluorescent cream and ultraviolet (UV) light that enables visualization of washing residue [13, 14]. When fluorescent dye is applied to the skin, UV light renders it luminous; thus, washing residue can be assessed by checking luminous areas after the skin has been washed. However, the evaluation methods used to assess the washing residue in these studies were subjective. Therefore, objective evaluations of washing residue on the feet are necessary.

Our study aimed to objectively examine the association between the presence of TP and the presence of washing residue on the feet. We examined the presence of washing residue between the toes and on the soles of the feet, where dermatophytes frequently adhere [15].

## 2. Materials and Methods

### 2.1. Study Design and Subjects

This cross-sectional study was conducted from January 2011 to December 2011. Diabetic patients who were admitted to the hospital or who had visited the Diabetic Foot Outpatient Clinic at the University of Tokyo Hospital were recruited for the study by a researcher. Participants received no incentive for participation. The inclusion criterion was diabetic patients who washed their own feet. Exclusion criteria were patients with foot ulcers, those who had changed their routine washing habits because of TP or other diseases, and those who had clinical findings that were characteristic of TP but had no direct microscopic evidence of a dermatophyte infection. The study protocol was approved by the Ethics Committee at the Graduate School of Medicine at The University of Tokyo (number 3228, [2]).

### 2.2. Study Procedure

First, photographs of participants' feet were taken, and skin and/or nail specimens were collected if there were any lesions such as scales, vesicles, cloudy nails, or nail thickening for detecting the presence or absence of TP. Second, the participants selected the items they wished to use for foot washing. Next, fluorescent cream was applied to the participants' feet, and photographs of the unwashed feet were taken under UV light. Lastly, photographs were taken after the participants had washed their own feet in the same way they did at home. The same researcher completed this process for all participants.

### 2.3. Data Collection

The presence of TP was confirmed if dermatophytes were detected using direct microscopy, and the absence of TP was defined as the clinical absence of lesions in the foot area. Although dermatophytes can theoretically parasitize the horny layer without any clinical signs, we defined a state in which there was no clinical evidence (i.e., scales and vesicles) for the absence of TP, because this condition does not cause secondary infections or ulcers. Participants who had clinical signs of TP but no microscopic evidence of a dermatophyte infection were excluded from the study, because this condition can represent a false-negative result. TP was categorized into three types: vesiculobullous, interdigital, and scaly or hyperkeratotic [16]. Tinea unguium was diagnosed when dermatophytes were detected within the nails. Familial TP was identified when participants had lived with any other person who had TP. Several standard items, including bar soap, body wash, towels, nylon towels, body sponges, and bath chairs, were available for the participants to select and to use while washing their feet in the same way as they did at home. We used a fluorescent cream (Spectro-pro Plus; Moraine Corporation, Tokyo, Japan) and a UV light (Hand Washing Checker BLB; Saraya Co., Ltd., Osaka, Japan) to detect the presence of washing residue. A researcher dispensed the fluorescent cream by pushing the nozzle of the bottle twice and applied it to the feet of each participant; then, using UV light in a dark room, the foot was checked to determine whether it was entirely covered with the fluorescent cream. Using a digital camera (IXY Digital 910IS; Canon Inc., Tokyo, Japan), photographs of the feet were obtained from a distance of 30 cm parallel to the soles or dorsa of the feet to provide baseline data. In addition, images of each of the areas between the toes were obtained by parting the toes with forceps. The same images were captured again under UV light after the feet had been washed. Foot washing was performed in a bathroom of a hospital ward.

The demographic characteristics of each patient were recorded, including their age, sex, and body mass index (BMI). Details of their diabetic-related factors, including the type of diabetes, duration of diabetes, and glycated hemoglobin (HbA1c) levels, were collected from the patients' charts. The ankle-brachial index was collected from the patients' charts, and when its value was ≤0.9, the patient was considered to have angiopathy [17]. Semmes-Weinstein 5.07 monofilaments were used for neurosensory testing as suggested by the International Working Group on Diabetic Foot [18], and vibratory perception was performed in accordance with the Japanese Diabetic Neuropathy Association manual [19]. Sensory neuropathy was diagnosed if the monofilament perception and/or the vibratory perception were abnormal. Patients' feet were assessed for the presence or absence of deformities, including bunions, bunionettes, claw toes, or hammer toes. Data relating to the histories of foot ulcers and amputations were collected from the patients' charts or during interviews with the patients. Participants were also interviewed about their foot-washing frequencies.

The washing residue was evaluated based on the images obtained from the anonymized participants. We defined washing residue as luminous areas under UV light. The soles of the feet were evaluated by calculating the fluorescence intensity reduction rate (FIRR). The outline of each sole was traced three times, and the mean fluorescence intensity was calculated using ImageJ software (National Institutes of Health, Bethesda, MD, USA). After deducting the fluorescence intensity before fluorescent cream application from that measured before and after foot washing, the FIRR was calculated using the following formula: FIRR (%) = (the fluorescence intensity before foot washing – the fluorescence intensity after foot washing)/the fluorescence intensity before foot washing × 100. We verified the reliability of this evaluation technique in a pilot study in which we calculated the FIRR for three foot-washing events that were performed by two healthy individuals. The intraclass correlation coefficient was 0.930. We also verified the discriminative validity of the technique by comparing the FIRR of two diabetic patients who had washed their feet thoroughly to that of two diabetic patients who had washed their feet insufficiently. The mean ± standard deviation (SD) FIRR of patients who had washed their feet thoroughly was significantly higher (81.4 ± 11.2%) than that of the patients who had washed their feet insufficiently (27.5 ± 1.6%) (*P* = 0.02). We confirmed that there was no difference in the FIRR between a patient with TP and an individual without TP when a researcher washed their feet in the same manner. The areas between the toes were evaluated according to the presence of fluorescent cream (PFC), because these areas are narrow and the shapes are complicated. Three researchers independently evaluated the images. When their decisions differed, a consensus was reached after discussion regarding the presence or absence of washing residues in order to maintain reliability.

### 2.4. Data Analysis

We compared patients who had TP with those who did not have TP with regard to the FIRR and PFC values using Fisher's exact or Student's *t*-tests. A *P* value of <0.05 was considered statistically significant. All the analyses were performed using Statistical Analysis System (SAS) software, version 9.3 (SAS Institute, Inc., Cary, NC, USA).

## 3. Results

Of the 145 candidates who had diabetes, 79 agreed to participate. Of these participants, 46 were excluded for the following reasons: one participant had a foot ulcer, 23 had clinical signs associated with TP but no microscopic evidence of a dermatophyte infection, 13 had cerebrovascular disorders that may have changed their washing performances, 6 had TP that may have affected their washing habits, and 3 had unclear foot images. Hence, 33 participants were included.

The mean ± SD age of the study participants was 62.2 ± 12.7 years. Twelve male participants (36.4%) were included. The mean ± SD body mass index was 25.9 ± 5.5, and 27 participants (81.8%) had type 2 diabetes. The mean ± SD diabetes duration was 16.3 ± 8.6 years, and the mean ± SD HbA1c value was 7.9% ± 1.3%. Six patients (18.2%) had foot deformities, none (0%) had histories of foot ulcers, and 1 (3.0%) had a history of foot amputation. Seventeen patients (51.5%) had TP (Table 1).

The BMI and the number of patients with type 2 diabetes were significantly higher in the group of patients with TP than in the group of patients without TP (*P* = 0.009 and *P* = 0.007, resp.) (Table 2).

**Figure 1:**
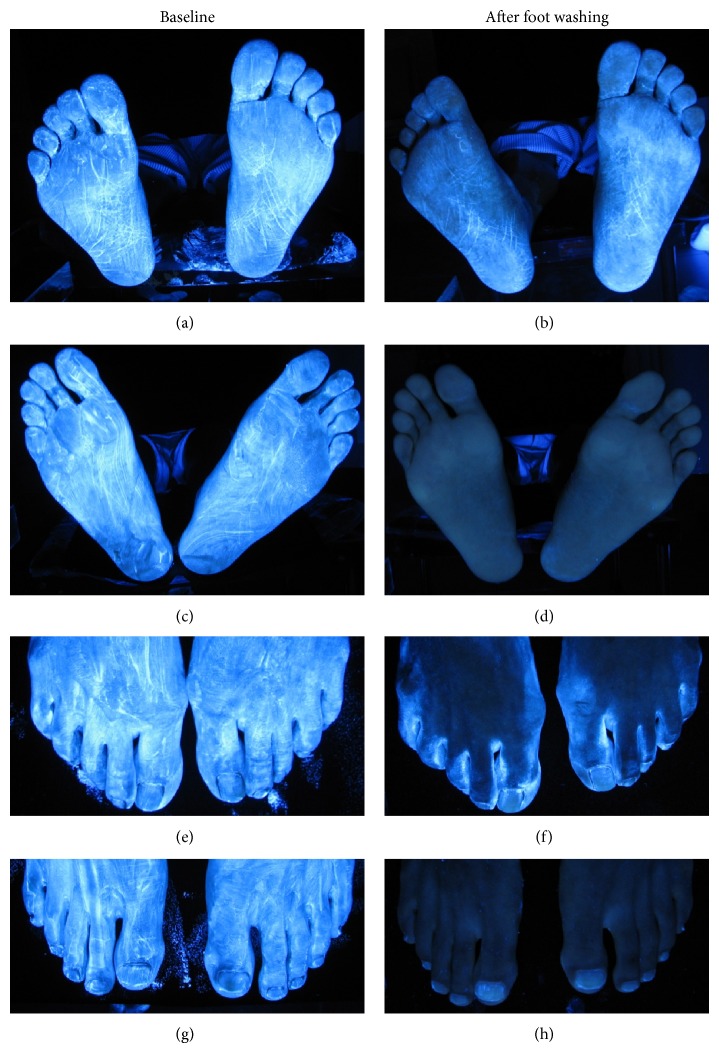
Representative images of patients' feet from both study groups under ultraviolet light before and after foot washing. The blue-white fluorescent areas indicate the presence of fluorescence from the residual cream, and the bluish-black areas represent the absence of fluorescence. (a) The soles of the participant's feet are infected with tinea pedis (TP) before foot washing. (b) The soles of the same participant's feet after foot washing. The fluorescence intensity reduction rate (FIRR) is 34.9%. (c) The soles of the participant's feet do not show TP infection before foot washing. (d) The soles of the same participant's feet after foot washing. The FIRR is 88.6%. (e) Areas between the toes of a participant with TP before foot washing. (f) Areas between the toes of the same participant after foot washing. This participant has residual cream. (g) Areas between the toes of a participant without TP infection before foot washing. (h) Areas between the toes of the same participant after foot washing. This participant has no residual cream. The nails are showing autofluorescence.

Representative images of patients' feet in both study groups captured under UV light before and after foot washing are shown in Figure 1. The mean ± SD wash time was 123.4 ± 64.2 s. The mean ± SD FIRR of the soles of the feet of the TP patients was significantly lower than that of the patients without TP (54.8% ± 23.3% versus 70.5% ± 13.6%; *P* = 0.025). The number of participants with PFC between the toes was significantly higher in the group of patients with TP than that in the group of patients without TP (64.7% versus 25.0%; *P* = 0.037) (Table 3).

No adverse events were associated with applying the fluorescent cream.

## 4. Discussion

This is the first study to objectively evaluate foot-washing residues using fluorescent cream and to compare the results between patients with TP and those without TP. Our study findings suggest that patients with TP are insufficiently skilled in foot washing, and they may be at a greater risk of contracting dermatophyte infections. Although diabetic patients are aware of the importance of foot washing, this study demonstrates the clinical value of foot-washing behavior in relation to TP prevention.

The fluorescent cream used in this study has an oil-in-water type of emulsion, which is easy to remove with detergents, because it contains an oily ingredient. Normally, dermatophytes are not one of the resident components of the skin flora, but they become part of transient skin flora by adhering to the skin. A previous study reported that 99.62% of the transient skin flora can be removed by hand washing alone using a detergent that did not contain antimicrobial ingredients [20]. Therefore, dermatophytes that adhere to the skin can be removed by washing with detergent, as determined by the UV results after applying fluorescent cream, in this study. Hence, washing residues observed in our study were considered to reflect residual dermatophytes. Since dermatophytes can traverse the surface of the horny layer after 12–24 h [7, 9], feet must be washed at least once daily. Washing feet daily is important, because they are not washed as frequently as the hands.

The correct hand-washing technique has been established for infection control [21]. Hand-washing education is conducted in schools and restaurants to prevent infections and food poisoning. However, with regard to foot washing for TP prevention in diabetic patients, no techniques have been established despite our previous study's findings, highlighting the importance of daily foot washing [10]. Furthermore, the current study findings, which showed that the amount of washing residue on the feet differed between patients with TP and those without TP, suggest that foot-washing behavior is an important factor in TP prevention. It is essential that the precise processes involved in foot washing be investigated in the future.

This study has several limitations. The results may be biased because the number of subjects in this study was small. A nationwide survey that included 198,353 diabetic subjects in Japan reported that the mean ± SD HbA1c level was 7.1% ± 1.4% and that the mean ± SD duration of diabetes was 10.5 ± 8.4 years [22]. In the current study, the HbA1c level was 0.4% higher than the level found in the nationwide survey, and the mean ± SD duration of diabetes was 5.8 years longer than that found in the nationwide survey. Although it is unclear how HbA1c levels and the duration of diabetes affect foot-washing residues, our findings must now be considered in the context of the nationwide diabetic population. Since the study participants received the same foot care education that all diabetic patients receive, we consider that our study findings can be extrapolated to all diabetic patients. The prevalence of TP among the study's participants was higher than the estimated prevalence of 20%, which is based on a previous study performed in Japan [23]. The higher prevalence of TP in this study may be explained by the fact that the participants visited the Diabetic Foot Outpatient Clinic with subjective symptoms relating to their feet. Since this study was not conducted to determine the prevalence of TP, we consider that the prevalence of TP among the study's participants had little effect on the findings. Given that this was a cross-sectional study, prospective studies are needed to reveal any causal relationships.

## 5. Conclusions

Our study demonstrated that diabetic patients with TP have habits that lead to insufficient foot washing compared to diabetic patients without TP.

The importance of foot washing needs to be emphasized through educational programs for diabetic patients. In addition, effective foot-washing techniques must be developed for diabetic patients. Further studies are needed to examine the correlations between TP and the factors of the foot-washing process in detail.

## Figures and Tables

**Table 1:** Patients' characteristics.

			*N* = 33
Age (years), mean ± SD			62.2 ± 12.7
Sex, *n* (%)	Male		12 (36.4)
	Female		21 (63.6)
BMI, mean ± SD		*n* = 30	25.9 ± 5.5
Type of diabetics, *n* (%)	Type 1		4 (12.1)
	Type 2		27 (81.8)
	Other		2 (6.1)
Diabetes duration (years), mean ± SD			16.3 ± 8.6
HbA1c (%), mean ± SD		*n* = 31	7.9 ± 1.3
Sensory neuropathy, *n* (%)	Yes		23 (69.7)
	No		10 (30.3)
Angiopathy, *n* (%)	Yes	*n* = 32	3 (9.4)
	No		29 (90.6)
Foot deformity^∗1^, *n* (%)	Yes		6 (18.2)
	No		27 (81.8)
History of foot ulcer, *n* (%)	Yes		0 (0.0)
	No		33 (100.0)
History of foot amputation, *n* (%)	Yes		1 (3.0)
	No		32 (97.0)
Tinea pedis, *n* (%)			17 (51.5)
Interdigit type			9 (27.3)
Vesicular type			4 (12.1)
Hyperkeratotic type			8 (24.2)
Tinea unguium, *n* (%)			9 (27.3)
Family with tinea pedis^∗2^, *n* (%)	Yes		18 (54.5)
	No		9 (27.3)
	Unknown		6 (18.2)
Foot-washing frequency, *n* (%)	Every day	*n* = 31	25 (80.6)
	Not every day		6 (19.4)

^∗1^Bunions, bunionettes, crow toes, hammer toes, and hollow foot.

^∗2^Inclusion of the family that the patients once lived with.

SD: standard deviation; BMI: body mass index; HbA1c: glycated hemoglobin.

**Table 2:** Characteristics of diabetic patients with or without tinea pedis.

			Patients with tinea pedis (*n* = 17)		Patients without tinea pedis (*n* = 16)	*P* value
Age (years), mean ± SD			63.4 ± 10.3		61.1 ± 15.2	0.613^1^
Sex, *n* (%)	Male		8 (47.1)		4 (25.0)	0.282^3^
	Female		9 (52.9)		12 (75.0)	
BMI, mean ± SD		*n* = 15	28.4 ± 5.8	*n* = 15	23.4 ± 3.9	0.009^1^
Type of diabetics, *n* (%)	Type 1		0 (0.0)		4 (25.0)	
	Type 2		17 (100.0)		10 (62.5)	0.007^3^
	Other		0 (0.0)		2 (12.5)	
Diabetes duration (years), mean ± SD			14.7 ± 7.9		17.9 ± 9.2	0.301^1^
HbA1c (%), mean ± SD		*n* = 16	7.7 ± 1.2	*n* = 15	8.0 ± 1.5	0.500^1^
Sensory neuropathy, *n* (%)	Yes		12 (70.6)		11 (68.7)	0.909^2^
	No		5 (29.4)		5 (31.3)	
Angiopathy, *n* (%)	Yes		1 (5.9)	*n* = 15	2 (13.3)	0.589^3^
	No		16 (94.1)		13 (86.7)	
Foot deformity, *n* (%)	Yes		3 (17.7)		3 (18.8)	1.000^3^
	No		14 (82.3)		13 (81.2)	
History of foot ulcer, *n* (%)	Yes		0 (0.0)		0 (0.0)	
	No		17 (100.0)		16 (100.0)	
History of foot amputation, *n* (%)	Yes		1 (5.9)		0 (0.0)	1.000^3^
	No		16 (94.1)		16 (100.0)	
Family with tinea pedis, *n* (%)	Yes	*n* = 14	10 (71.4)	*n* = 13	8 (61.5)	0.695^3^
	No		4 (28.6)		5 (38.5)	
Foot-washing frequency, *n* (%)	Every day	*n* = 16	13 (81.2)	*n* = 15	12 (80.0)	1.000^3^
	Not every day		3 (18.8)		3 (20.0)	

^1^ *t*-test; ^2^ *χ* ^2^-test; ^3^Fisher's exact test.

SD: standard deviation; BMI: body mass index; HbA1c: glycated hemoglobin.

**Table 3:** Washing residue on the feet.

	Patients with tinea pedis (*n* = 17)	Patients without tinea pedis (*n* = 16)	*P* value
Sole			
Fluorescence intensity reduction rate (%), mean ± SD	54.8 ± 23.3	70.5 ± 13.6	0.025^†^
Between the toes			
Presence of fluorescent cream, *n* (%)			
Yes	11 (64.7)	4 (25.0)	0.037^‡^
No	6 (35.3)	12 (75.0)	

^†^ *t*-test; ^‡^Fisher's exact test.

SD: standard deviation.
